# Non-Fat Yogurt Fortified with Whey Protein Isolate: Physicochemical, Rheological, and Microstructural Properties

**DOI:** 10.3390/foods10081762

**Published:** 2021-07-30

**Authors:** Mahmood A. Hashim, Liudmila A. Nadtochii, Mariam B. Muradova, Alena V. Proskura, Khalid A. Alsaleem, Ahmed R. A. Hammam

**Affiliations:** 1 Faculty of Biotechnologies (BioTech), ITMO University, 197101 Saint Petersburg, Russia; l_tochka@itmo.ru (L.A.N.); mari.muradova1996@gmail.com (M.B.M.); pav060695@mail.ru (A.V.P.); 2 Agricultural Research Centre, Food Technology Research Institute, Giza 85871, Egypt; 3 Dairy and Food Science Department, South Dakota State University, Brookings, SD 57007, USA; Khalid.alsaleem@sdstate.edu (K.A.A.); ahmed.hammam@sdstate.edu (A.R.A.H.); 4 Department of Food Science and Human Nutrition, College of Agriculture and Veterinary Medicine, Qassim University, Buraydah 51452, Saudi Arabia; 5 Dairy Science Department, Faculty of Agriculture, Assiut University, Assiut 71526, Egypt

**Keywords:** non-fat yogurt, whey protein, water holding capacity, rheology, texture, microstructure, sensory properties

## Abstract

The demand for low- and non-fat products has recently increased due to the health problems, such as obesity, diabetes, and cardiovascular diseases, that have resulted from high-fat products. However, the reduction in fat can affect the quality of products adversely. The objective of this work was to explore the potential of whey protein isolate (WPI) in improving the quality of non-fat yogurt prepared using skim milk powder (SMP). Yogurt mixes (standardized at 14% total solids) were formulated using SMP as a milk base enriched with WPI. The SMP was replaced by WPI in the yogurt mixes at a rate of 3, 5, 7, and 9%. Full-fat and non-fat set-style yogurts were prepared from whole milk and skim milk, respectively, as controls. Yogurts were fermented at 43 °C to get a pH of 4.6 and stored at 4 °C for the next day. The texture, microstructure, rheological characteristics, and sensory properties of the yogurt samples were studied. The incorporation of WPI increased the water holding capacity to 50% as compared to the non-fat control. This improved the rheological properties while the yogurt viscosity increased in direct proportion with increasing the WPI. The firmness of yogurt was inversely proportional to the increase in WPI, which resulted in 180 g firmness when 9% WPI was added to the non-fat yogurt formulations. Yogurts’ microstructure improved by the addition of WPI. The non-fat yogurt incorporated with 3 and 7% WPI had comparable sensory and textural characteristics to the full-fat yogurt. WPI can be used as a fat replacer to develop low-fat yogurt with desired features. WPI may be a natural and economical ingredient for producing low- and non-fat fermented dairy food products.

## 1. Introduction

The extreme consumption of food nutrients (such as fats and carbohydrates) has become a significant problem. According to the formula of balanced nutrition, the daily demand of the human organism for fats is 102 g; however, an analysis of the macronutrient status of Russians shows that this value has increased two-fold. The World Health Organization (WHO) reported an increase in the number of people with progressing civilizational diseases, such as obesity, diabetes mellitus, and cardiovascular diseases [1]. Consequently, the production of reduced or low-fat dairy products has significantly increased around the world. Sales of such products have elevated due to their nutritional values with fewer health problems [2].

The manufacture of yogurt from partial skim milk or skim milk has resulted in an undesirable flavor, body and texture, as well as rheological and functional properties. Set yogurt manufacturers are frequently challenged with the fracture of the gel and wheying-off because this type of yogurt is fermented in its commercial package. The most popular trend to overcome these challenges is the utilization of milk modified with skim milk powder (SMP). The replacement of milk with dry dairy products may allow the obtaining of a low-fat set yogurt with the same viscosity and less syneresis to satisfy consumers at a lower industrial cost. Milk proteins (consisting of 80% casein and 20% whey protein) are essential components of dairy products and characterized by their multifunctional properties. These components have the possibility to replace fat or enhance the structure of fat-reduced yogurts due to their unique texturing properties [3].

The addition of whey protein concentrate (WPC) and whey protein isolate (WPI) ingredients has improved water binding or water holding capacity (WHC) [4], foaming [5], gelling [6], and emulsifying properties [7] in food systems. Whey protein products have been used as ingredients in a wide range of food applications due to their high nutritional, biological, and unique functional properties. Whey protein displays various performance properties similar to fat and can be used as a fat replacer [8,9,10]. The microstructure of yogurt has been studied extensively using scanning electron microscopy (SEM) and transmission electron microscopy. The results of many of these studies have been reviewed [11]. Electron microscopy shows that yogurt consists of a casein micelle network that associates to form a gel-like structure. This protein network has interstitial spaces that contain the liquid phase as well as larger void spaces containing starter bacteria. The structure of the protein matrix varies, depending on protein content, heat treatment of the mix [12,13], and the presence or absence of milk fat, thickening agents (stabilizers), and bacterial exopolysaccharides [11,14,15]. The objective of the present work is to study the possibility of using WPI as an ingredient in making non-fat set yogurt and study the rheological, structural, and sensory properties.

## 2. Materials and Methods

### 2.1. Manufacture of Yogurt

WPI (protein 87.8%, moisture 4.6%, fat 0.7%), SMP (protein 28%, moisture 5%, fat 1.5%), and whole milk powder (WMP: protein 26.5%, moisture 5%, fat 26.5%) were used for yogurt formulations and obtained from Fonterra, New Zealand (supplied by Unifood Company, Saint Petersburg, Russia). The total solid (TS) of all yogurt samples was standardized at 14%. Two control samples were utilized for comparison reasons as follows; Control-1 or full-fat yogurt (FFY) which was produced from WMP and Control-2 or non-fat yogurt (NFY) that was manufactured from SMP. The SMP was replaced by WPI at a rate of 3% (NFYWP-3), 5% (NFYWP-5), 7% (NFYWP-7), and 9% (NFYWP-9), while maintaining 14% TS. All ingredients (powders and water) were mixed at room temperature for 15 min using a magnetic stirrer at a scale of 150 g for each formulation. All prepared solutions were refrigerated at 7 ± 1 °C overnight to allow full hydration ability of the dispersed powder and to establish mineral or salt equilibrium, which can increase the viscosity of yogurt mixes.

The yogurt was manufactured as described in previous studies [16,17] with some modifications. The prepared mixes were pasteurized (85 °C/30 min), cooled to 43 °C, and then inoculated with 0.3% *w/w* freeze-dried yogurt starter culture (YF-L812, Chr. Hansen, Denmark) that contained *Streptococcus thermophilus* and *Lactobacillus delbrueckii* ssp. *bulgaricus*. The pH was monitored every 30 min until fermentation was completed at a pH of 4.6, which took around 5 h. The yogurt was cooled in a refrigerator overnight until further analyses. This experiment was replicated three times.

### 2.2. Viscosity of Yogurt Mixes

Brookfield viscometer (BROOKFIELD DVII, Middleboro, MA, USA) was used for measuring the viscosity of yogurt mixes with spindle No 5 at 50 rpm and sample temperature of 3 ± 1 °C. The viscosity values were read from the instrument as centipoise (cP) as described in Izadi’s study [18].

### 2.3. Water Holding Capacity

The WHC of yogurts was measured [19,20]. Approximately 25 g of yogurt was weighed in a 40-mL tube and centrifuged (Sigma 3-16L GmbH) at 15,000× *g* for 15 min at 25 °C. The supernatant layer was removed and then yogurt samples were weighed. This analysis was performed in triplicate. The WHC was expressed as g pellet per 100 g yogurt.

$$\mathrm{WHC}\;(\%)=\frac{\mathrm{weight}\;\mathrm{of}\;\mathrm{the}\;\mathrm{remaining}\;\mathrm{after}\;\mathrm{centrifugation}\;\left(g\right)}{\mathrm{weight}\;\mathrm{of}\;\mathrm{the}\;\mathrm{sample}\;\left(g\right)}\;\times \;100$$

### 2.4. Firmness of Yogurt

The yogurt was prepared in glass containers (4 cm diameter and 7 cm length) before measuring the firmness (g). The firmness of the yogurt was measured using a universal information measuring system created based on a domestic texture analyzer (Structurometer ST2). The maximum force required to penetrate the gel was taken as a measure of the relative gel strength. We used method of Bloom No 2 named “Determination of the strength of gelatin” (International Standards: BSI: BS 757-1975; AFNOR: NF V59-001; ISO 9665). A texture profile analysis (TPA) model was performed with a penetration distance of 4 mm using a cylindrical probe (15 mm diameter, 20 mm height). The speed of the probe was one mm/s with a trigger of 7 g. The analyzer was connected to a computer that documented data via a software program [21,22]. The firmness of the yogurt samples was estimated and defined as the force necessary to attain a given deformation or the force necessary to reach the maximum depth. Each test was replicated four times.

### 2.5. The Rheological Characteristics of Yogurt Samples

The apparent viscosity of yogurt was measured using a rheometer (Rheotest RN 4.1, Rheotest Medingen GmbH, Ottendorf-Okrilla, Germany). The samples of required volume were loaded to the rheometer and the shear was held using a coaxial cylinder. This consists of a stationary measuring cup and a cylindrical rotor, which is placed on the cup. To study the behavior of liquids at different temperatures, thermostated vessels are available.

All rheological measurements were carried out at 10 °C (temperature was regulated by bath model Julabo^®^ refrigerated and heating circulators FP50-HP). A sample of 15 g was loaded to the apparatus, and the steady shear viscosity of the yogurt samples was measured as a function of shear rates from 0.1 to 10 s^−1^ (in the forward direction). To assess the ability of yogurt to recover their structure after mechanical impact, the samples were left to rest for 15 min, and then, again, they were subjected to mechanical stress rates from 10 to 0.1 s^−1^ (in reverse). Between the two curves (in direct and in opposite) hysteresis loop area is inversely proportional to the ability of yogurt to rebuild its gel structure after undergoing shear, so it is a measurement of the quality of the product. The rheological properties were examined three times.

### 2.6. Microstructure of Yogurt

Electron micrographs of the yogurt samples were obtained with SEM (JSM-6390LA, JEOL Ltd., Tokyo, Japan). Samples were prepared as mentioned in previous studies with some modifications [11]. Sections of yogurt samples were fractured with a blade and fragments were mounted on aluminum SEM stubs by conductive silver cement. They were coated with gold by vacuum evaporation in a nitrogen atmosphere at 66 mPa. SEM was operated at 15 kV, and all samples were studied at a larger resolution for better characterization of the sample microstructure.

### 2.7. Organoleptic Properties of Yogurt

The quality of the final yogurts was assessed using the analysis of the sense-perception: sight, smell, touch, and taste. All 6 samples (FFY, NFY, NFYWP-3, NFYWP-5, NFYWP-7, and NYWP-9) were exhibited to the panelists. Participants of 12 members (6 women and 6 men, aged 22 to 40 years) were selected with relevant experience and knowledge in the sensory evaluation of dairy products. The invited panelists were from the Faculty of Biotechnology. Sensory descriptors of appearance (30), flavor (10), and texture (60) were evaluated by the sensory panels [23] to give an overall score of 100 points. The sensory characteristics were also repeated three times.

### 2.8. Statistical Analysis

Analysis of variance (ANOVA) as well as the average and standard error were carried out using SPSS computer-based program. Mean separation was completed when significant differences were detected at *p* < 0.05.

## 3. Results and Discussion

### 3.1. Viscosity of Yogurt Mixes

The viscosity of yogurt mixes steadily increased with the increase in WPI concentration (Table 1). A significant difference (*p* < 0.05) was detected in the yogurt samples, where FFY, NFY, NFYWPI-3, NFYWPI-5, NFYWPI-7, and NFYWPI-9 had 2.88 ± 0.34, 1.84 ± 0.45, 2.12 ± 0.73, 2.49 ± 0.32, 2.31 ± 0.51, and 2.49 ± 0.24 cP, respectively. The increment in the viscosity could be due to the increase in protein content from whey proteins, which have a positive effect on milk viscosity and their ability to bind water [24]. During the manufacturing of yogurt, heat treatment forms a complex between casein and whey protein and this complex formation depends on the presence of whey proteins in mixes [25]. On the other hand, applied heat treatment defines the amount of α-lactalbumin that associates with casein micelle and, thus, influences the hydrophilic properties and hydration of casein micelle at pH of 4.6 and, therefore, the rheological properties of acid casein gel [26].

### 3.2. Water Holding Capacity (WHC)

The WHC is one of the most important factors used to evaluate set yogurt. WHC is enhanced by denatured whey proteins that result in a reduction of whey syneresis, which is an important factor that negatively impacts its quality [27].

**Figure 1:**
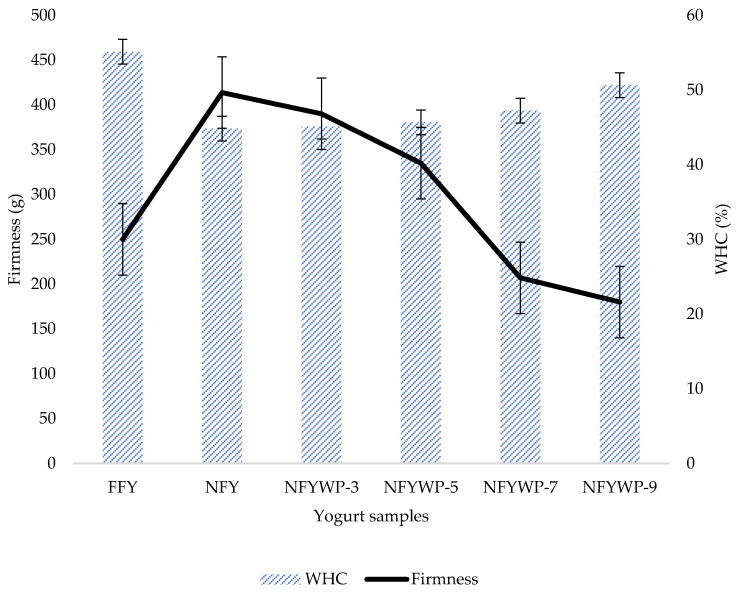
The firmness (g) and water holding capacity (WHC) of yogurt samples. FFY = full-fat yogurt produced from whole milk powder (WMP); NFY = non-fat yogurt produced from skim milk powder (SMP); NFYWPI-3 = SMP was replaced by 3% WPI; NFYWP-5 = SMP was replaced by 5% WPI; NFYWP-7 = SMP was replaced by 7% WPI; NFYWP-9 = SMP was replaced by 9% WPI.

WHC gradually increased with WPI substitution in NFYs (Figure 1). Significant differences (*p* < 0.05) were detected in the percentage of WHC as FFY (55.21 ± 0.31%), NFY (44.83 ± 0.07%), NFYWPI-3 (45.09 ± 0.14%), NFYWPI-5 (45.67 ± 0.25%), NFYWPI-7 (47.23 ± 0.17%), and NFYWPI-9 (50.63 ± 0.35%). The presence of fat significantly influenced the WHC as described in FFY and NFY; besides, the WHC gradually increased with the increase in the concentration of WPI in NFYs. FFY had the highest WHC regarding its fat ability to bind water and the role of fat in the formation of the gel networks [19]. Fortification of NFY with WPI significantly increased the WHC of yogurt samples, which was in agreement with a previous study [27]. The increase in WHC may result from the denaturation of whey proteins and the interactions between denatured whey protein and κ-caseins that can increase the formation of a homogeneous porous structure in which free water was immobilized and encapsulated [3,28].

### 3.3. The Texture of Yogurt

Substitution of SMP with WPI significantly (*p* < 0.05) affected the texture of the yogurt samples. This technique is based on the determination of the loading force parameter. Changes in the load force on the indenter depending on the depth of its introduction into yogurt samples are presented in Figure 1. It is considered that the NFY exhibited the highest firmness (414 g), in contrast NFYWPI-9 showed the highest deformation and the lowest firmness (180 g). Increasing WPI significantly decreased the firmness of yogurts (Figure 1). Thus the firmness of NFY with 3, 5, and 7% WPI was 390, 335, 207 g, respectively. Non-fat yogurt exhibited the highest firmness aligned with the lowest deformation, and that may be due to the absence of fat, which gives a strong and interconnected microstructure, as described in previous studies [29,30]. Those studies mentioned that the microstructure of the gel of fermented milk products consists of: (a) protein matrices composed of casein micelle chains and clusters; (b) attachments of denatured β-lactoglobulin that forms a complex with κ-casein; (c) fat globules (if present in the milk base) embedded in the casein particles; (d) annulled spaces within the protein matrices. The firmness of yogurt depends on the type and the content of protein as well as the total solids content [31,32,33]. Increasing the substitution of SMP with WPI led to a limited decreasing in the firmness of the yogurts. However, the addition of greater WPI led to a decrease in the firmness, and yogurts became more viscous compared with full-fat yogurt.

### 3.4. Rheological Properties of Yogurt Samples

**Figure 2:**
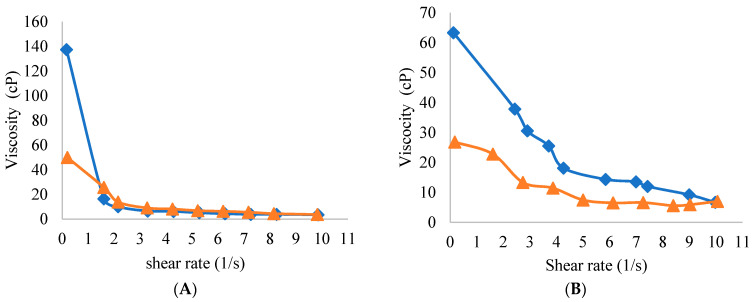

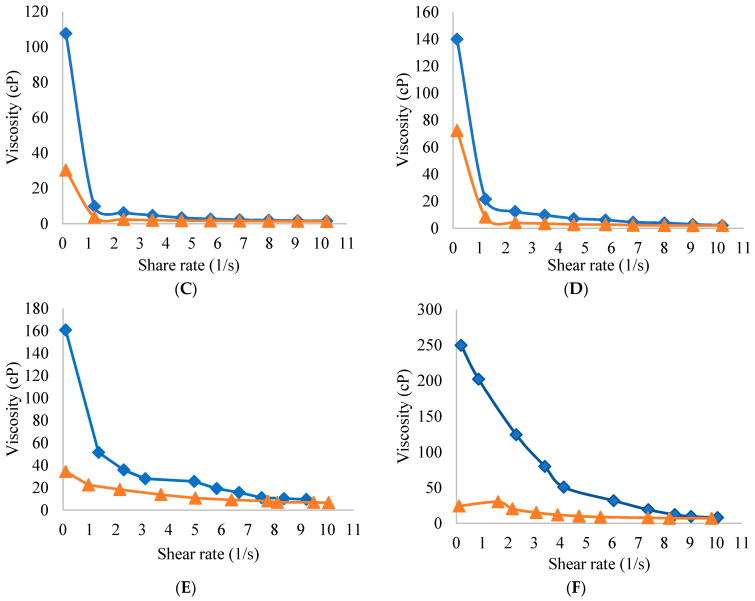
The apparent viscosity properties of yogurt samples: in straight/forward (♦); in opposite/reverse (▲). (**A**) FFY = full-fat yogurt produced from whole milk powder (WMP); (**B**) NFY = non-fat yogurt produced from skim milk powder (SMP); (**C**) NFYWPI-3 = SMP was replaced by 3% WPI; (**D**) NFYWP-5 = SMP was replaced by 5% WPI; (**E**) NFYWP-7 = SMP was replaced by 7% WPI; (**F**) NFYWP-9 = SMP was replaced by 9% WPI.

All yogurt samples expectedly exhibited pseudoplastic (shear-thinning) behavior and were characterized as non newtonian fluid as shown in Figure 2A–F. As a result, the relationship between shear rate and the resulting stress is not as linear as it is for a newtonian fluid. As shown in Figure 2A,B, FFY and NFY significantly differed (*p* < 0.05) where FFY samples illustrated higher apparent viscosity in the straight direction with shearing than NFY. In addition, NFY samples showed the lowest apparent viscosity with shearing in comparison with other yogurts. Apparent viscosity for yogurts fortified with WPI showed significant differences in the straight direction with shearing, where the viscosity gradually increased with increasing WPI. Yogurts fortified with 5 and 7% WPI scored the most acceptable values for the apparent viscosity and showed appropriate viscous structure and as fatty-like as the full-fat yogurt. Yogurt with 9% WPI gave the highest value for the apparent viscosity. The hysteresis loop area of NFYWPI-7 and NFYWPI-9 were significantly bigger than the area of NFYWPI-3 and NFYWPI-5. The 3 and 5% WPI treatments were similar to FFY, which means that these yogurt samples could rebuild their gel structure after undergoing shear compared to NFYWPI-7 and NFYWPI-9. Therefore, the reduction in the apparent viscosity of yogurts with increasing shear rate was a result of the destruction of the interactions. The apparent viscosity of yogurt was significantly affected by the fortification of WPI, indicating a possible gradual increase in the apparent viscosity with the increase in the addition of WPI. It was demonstrated that WPI can lead to the formation of a stronger protein network, as it may intricate with protein aggregates via hydrogen bonds, further increasing the viscosity due to its water retention capacity [30]. Similar results were reported in another study [34], which found high thickness in yogurts of the type labneh, with a higher protein content. Another experiment demonstrated that higher whey protein concentration can result in higher yield stress and viscosity in yogurt by forming a denser and more intense network [35].

Yogurt samples exhibited thixotropic characteristics when shear forces disrupted the microstructure of materials. This structure partially or fully recovered when the material was quiescent and the structure of thixotropic yogurts partially or fully rebuilt after the removal of shear, resulting in an increase in viscosity up to the original viscosity after the yogurt was left at rest. Researchers associated that the thixotropy in heated whey proteins suspension was attributed to the particle breakage, or full breakage of disulfide bonds, and van der Waals, ionic and hydrophobic interactions between the protein particles [36]. Particle breakage and full breakage of weak bonds between particles could also cause thixotropy in yogurt. Further, the driving force for thixotropic behavior is the competition between structural breakdown due to applied force and structural buildup due to in-flo collisions and Brownian motion [37]. The area of a yogurt sample’s hysteresis loop is inversely proportional to its ability to rebuild its gel structure after undergoing shear. NFY samples showed the lowest apparent viscosity with shearing in comparison with other yogurts and this may be due to the presence of fat in FFY and its absence in NFY. These results are in agreement with those stated: that the decreased fat content in yogurt can result in low viscosity due to the decrease in total milk solids [38].

**Figure 3:**
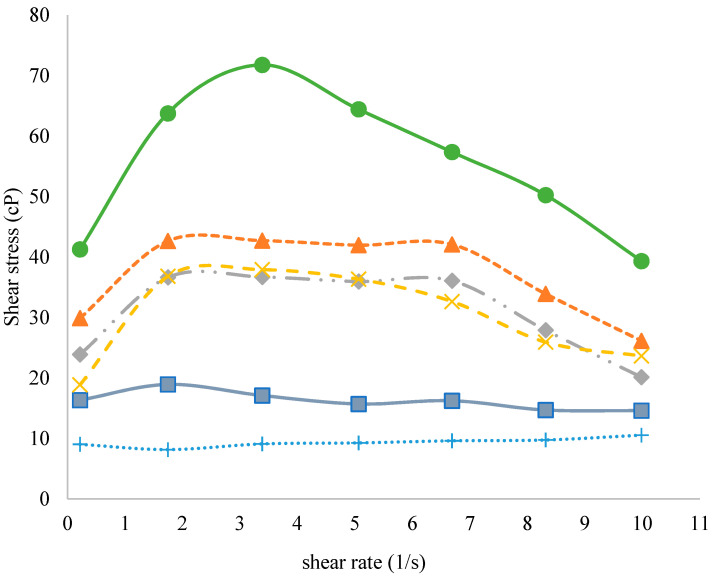
The rheological characteristics of yogurt samples. FFY (●) = full-fat yogurt produced from whole milk powder (WMP); NFY (■) = non-fat yogurt produced from skim milk powder (SMP); NFYWPI-3 (▲) = SMP was replaced by 3% WPI; NFYWP-5 (♦) = SMP was replaced by 5% WPI; NFYWP-7 (x) = SMP was replaced by 7% WPI; NFYWP-9 (+) = SMP was replaced by 9% WPI.

Shear stress versus shear rate relationships for fat-free yogurt, fat-free yogurt with WPI, and whole-fat yogurt are shown in Figure 3. FFY exhibited the highest shear stress values (*p* < 0.05) in comparison with other samples; while NFY and NFYWPI-9 illustrated the lowest shear stress values. This may be due to the absence of fat in NFY, yet for NFYWP-9, this may be due to the high content of water that was absorbed by the denatured whey protein isolate, which decreased the shear stress. Besides, the non-fat yogurts with 3, 5, and 7% WPI exhibited higher shear stress values than non-fat yogurt and less than full-fat yogurt. This indicated a comparatively thicker structure of the three yogurts with WPI added. The shear-thinning behavior or pseudoplastic was expected in yogurts because the texture of fermented milk products is affected by weak physical bonds, and electrostatic and hydrophobic interactions [39]. It is more appropriate to measure shear stress at an increasing shear rate rather than record single point measurements [40]. The existence of yield stress is generally linked to the presence of an interactive or cross-linked structure [41]. Adding WPI affects the increase in the shear stress of non-fat yogurts; in addition, the strength of protein-protein bonds, the number of bonds per cross-section of the strand, relaxation times for the network bonds, and the orientation of strands in the matrix all contribute to the yield properties of gels [42]. The rheological behavior of yogurt is influenced by a three-dimensional network formed by protein [43]. The enhanced milk protein content facilitated the yogurts to form strong protein-protein bonds [44].

### 3.5. Microstructure of Yogurt

**Figure 4:**
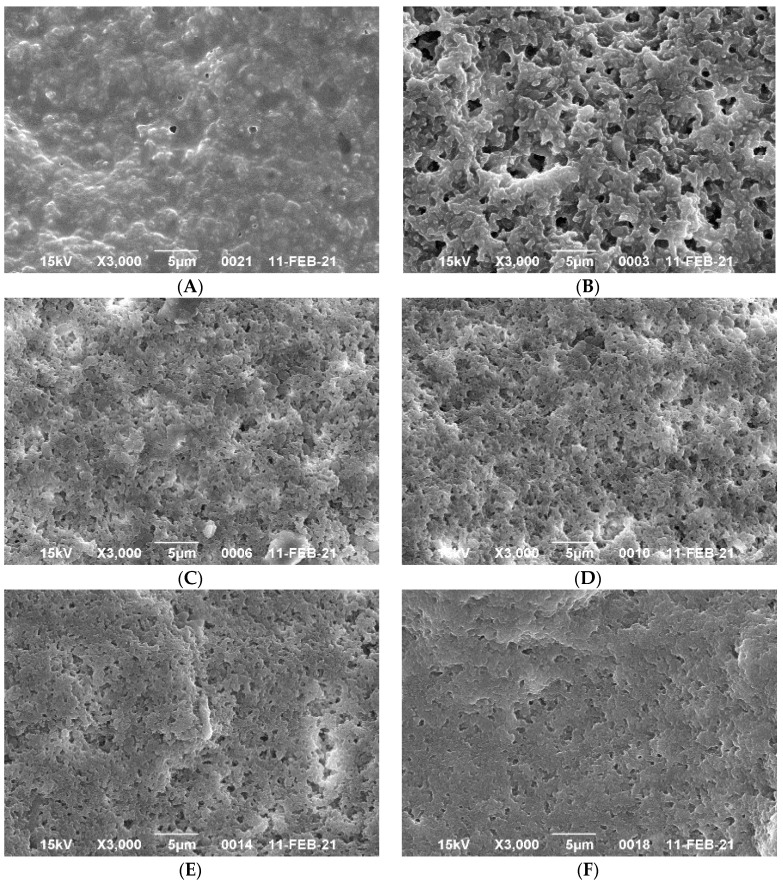
Scanning Electron Microscopy (SEM) photographs of yogurt samples. (**A**) FFY = full-fat yogurt produced from whole milk powder (WMP); (**B**) NFY = non-fat yogurt produced from skim milk powder (SMP); (**C**) NFYWPI-3 = SMP was replaced by 3% WPI; (**D**) NFYWP-5 = SMP was replaced by 5% WPI; (**E**) NFYWP-7 = SMP was replaced by 7% WPI; (**F**) NFYWP-9 = SMP was replaced by 9% WPI.

The SEM micrographs show the particles and pore size of different yogurt samples (Figure 4). The microstructure of control yogurt samples with different fat content showed different protein network structures. The fat globules were embedded in the protein network occasionally. The microstructure of FFY was composed of the network structure of casein gel and the interaction between fat globules and protein networks (Figure 4A). FFY exhibited the densest structure compared with other yogurts. The microstructure of NFY (Figure 4B) was the lowest density than other yogurts with more open spaces than CFY. The non-fat yogurts samples fortified with WPI had a denser network microstructure, and less space between protein clusters than the control non-fat NFY.Increasing WPI fortified with non-fat yogurts led to the structure becoming more condense and more compact as shown in Figure 4C–F. The casein micelle clusters of NFY were smaller than that of FFY, probably due to the smaller number of fat globules linking protein agents [28,45]. The yogurt samples with WPI had a denser network structure and a lower network in contrast with the control NFY that may be due to the addition of WPI. The yogurt had a denser casein network, which may be due to the chains formed by the casein micelle bundles connected to the denaturated WPI via disulfide bonds which became free. It is also probable that β-lactoglobulin, the significant fraction of WPI, reacted with κ-casein of the casein micelles during heat treatment and formed an insoluble complex. This would explain the accumulation of protein and the participation and formation of the linkage between the micelles, especially in Figure 4E,F. The increment in addition WPI significantly illustrated more condensation in the micrographs as shown in Figure 4C–F.

### 3.6. Sensory Properties of Yogurt

According to the results of hedonic analysis, it was concluded that significant differences were observed among yogurts with WPI that differed slightly (*p* < 0.05) from control full-fat and non-fat yogurt in terms of structure. Table 2 shows that the FFY sample showed the most acceptable organoleptic characteristics compared to the rest of the samples. The NFY sample exhibited the best results compared to samples with WPI in terms of appearance and taste, but yogurts fortified with WPI exhibited higher scores in the structure. Addition of WPI markedly improved the body and texture (3 and 5% WPI), and flavor (3% WPI) of yogurt samples compared to control samples. However, the highest (*p* < 0.05) score was recorded in NFYWPI-5, and it was accepted as the reference FFY. With increasing WPI in the samples NFYWP-7 and 9, the texture became more viscous and less acceptable for most panelists.

The NFY sample scored the lowest values, which could be due to the absence of fat and the low water content as well as the greater firmness than samples with WPI. These results indicate that the use of WPI could improve the sensory properties of yogurt. The proportion of whey proteins and caseins in formulations is critical to simultaneously improving yogurt sensorial characteristics [46]. In addition, whey protein acts in a way similar to integrated fat globules so that the quality of some dairy products, particularly with low-fat content, is improved. Therefore, treating set yogurts with whey protein improves nutrient value, yield, and functional and organoleptic properties [47].

## 4. Conclusions

The substitution of WPI with SMP in yogurt mixes produced yogurt gels with high stiffness, high WHC, and a more acceptable structure. These conditions contributed to creating a complex gel network involving numerous aggregating particles, as well as to increase the number and strength of contact points, which were responsible for gel rigidity and resistance to deformation. The incorporation of WPI proved of particular importance to replacing fat and enhancing the structure of fat-reduced yogurts regarding their texturing properties. This study suggests that the substitution of milk proteins with the addition of WPI in the reduced fat and non-fat yogurts improved their quality but this depends on improvement of the texture and physical characteristics. Thus, set yogurts fortified with WPIs were softer and suffered less syneresis than control yogurts. WPIs may be considered as an added value in yogurt production. Further studies on the sensory properties and storage behavior of such gels are required to establish the full potential of whey proteins in set-type yogurts.

## Figures and Tables

**Table 1:** The viscosity of yogurt mixes measured in different treatments.

Treatments ^1^	Viscosity (cP)
FFY	2.88 ± 0.34 ^a^
NFY	1.84 ± 0.45 ^c^
NFYWPI-3	2.12 ± 0.73 ^b^
NFYWPI-5	2.49 ± 0.32 ^ab^
NFYWPI-7	2.31 ± 0.51 ^b^
NFYWPI-9	2.49 ± 0.24 ^ab^

^a–c^ Means in the same column not sharing a common superscript are different (*p* < 0.05). ^1^ FFY = full-fat yogurt produced from whole milk powder (WMP); NFY = non-fat yogurt produced from skim milk powder (SMP); NFYWPI-3 = SMP was replaced by 3% WPI; NFYWP-5 = SMP was replaced by 5% WPI; NFYWP-7 = SMP was replaced by 7% WPI; NFYWP-9 = SMP was replaced by 9% WPI.

**Table 2:** The sensory properties of yogurt made using whey protein isolate (WPI).

Treatments ^1^	Appearance (30)	Texture (60)	Flavor (10)	Total (100)
FFY	27.81 ± 0.31	57.73 ± 0.25 ^a^	9.13 ± 0.14 ^a^	94.67 ± 0.34 ^a^
NFY	28.53 ± 0.62	54.24 ± 0.17 ^b^	8.76 ± 0.35 ^ab^	91.53 ± 0.65 ^b^
NFYWPI-3	28.01 ± 0.17	56.06 ± 0.73 ^ab^	8.87 ± 0.82 ^ab^	92.94 ± 0.72 ^ab^
NFYWPI-5	27.93 ± 0.39	56.85 ± 0.81 ^ab^	8.19 ± 0.94 ^b^	92.97 ± 0.19 ^ab^
NFYWPI-7	27.87 ± 0.64	53.84 ± 0.59 ^c^	8.65 ± 0.31 ^ab^	90.36 ± 0.83 ^c^
NFYWPI-9	26.84 ± 0.51	52.65 ± 0.38 ^c^	8.31 ± 0.57 ^b^	87.80 ± 0.87 ^d^

^a–d^ Means in the same column not sharing a common superscript are different (*p* < 0.05). ^1^ FFY = full-fat yogurt produced from whole milk powder (WMP); NFY = non-fat yogurt produced from skim milk powder (SMP); NFYWPI-3 = SMP was replaced by 3% WPI; NFYWP-5 = SMP was replaced by 5% WPI; NFYWP-7 = SMP was replaced by 7% WPI; NFYWP-9 = SMP was replaced by 9% WPI.

## Data Availability

Data sharing not applicable.

## References

[1] Smirnova I. (2014). Current Trends in Nonfat Dairy Production. Foods Raw Mater..

[2] Molina E., Dolores Álvarez M., Ramos M., Olano A., López-Fandiño R. (2000). Use of high-pressure-treated milk for the production of reduced-fat cheese. Int. Dairy J..

[3] Lesme H., Rannou C., Famelart M.-H., Bouhallab S., Prost C. (2020). Yogurts enriched with milk proteins: Texture properties, aroma release and sensory perception. Trends Food Sci. Technol..

[4] Kontopidis G., Holt C., Sawyer L. (2004). Invited Review: β-Lactoglobulin: Binding Properties, Structure, and Function. J. Dairy Sci..

[5] Bals A., Kulozik U. (2003). Effect of pre-heating on the foaming properties of whey protein isolate using a membrane foaming apparatus. Int. Dairy J..

[6] Kerstens S., Murray B., Dickinson E. (2005). Confocal microscopy of heat-induced aggregation and gelation of β-lactoglobulin in presence of non-ionic surfactant. Food Hydrocoll..

[7] Leman J., Smoczyñski M., Dolgañ T., Dziuba Z. (2005). Fractal analysis of structure of cow and goat β-lactoglobulin preparations. J. Food Sci. Technol..

[8] Korhonen H., Pihlanto-Leppälä A., Rantamäki P., Tupasela T. (1998). The functional and biological properties of whey proteins: Prospects for the development of functional foods. Agric. Food Sci..

[9] García-Garibay M., Jiménez-Guzmán J., Hernández-Sánchez H., Custavo V.J., Chansen E.A. (2008). Whey Proteins: Bioengineering and Health. Food Engineering Integrated Approoches.

[10] Vidigal M.C.T.R., Minim V.P.R., Ramos A.M., Ceresino E.B., Diniz M.D.M.S., Camilloto G.P., Minim L.A. (2012). Effect of whey protein concentrate on texture of fat-free desserts: Sensory and instrumental measurements. Food Sci. Technol..

[11] Modler H.W., Kalab M. (1983). Microstructure of Yogurt Stabilized with Milk Proteins. J. Dairy Sci..

[12] Harwalkar V.R., Kalab M. (1986). Relationship between microstructure and susceptibility to syneresis in yoghurt made from reconstituted non fat dry milk. Food Microstruct..

[13] Kalab M., Emmons D.B., Sargant A.G. (1976). Milk gel structure V. Microstructure of yoghurt as related to the heating of milk. Milchwiss. Milk Sci. Int..

[14] Kalab M., Emmons D.B., Sargant A.G. (1975). Milk-gel structure. IV. Microstructure of yoghurts in relation to the presence of thickening agents. J. Dairy Res..

[15] Teggatz J.A., Morris H.A. (1990). Changes in the rheology and microstructure of ropy yogurt during shearing. Food Struct..

[16] Kamel D.G., Hammam A.R.A., Alsaleem K.A., Osman D.M. (2021). Addition of inulin to probiotic yogurt: Viability of probiotic bacteria (Bifidobacterium bifidum) and sensory characteristics. Food Sci. Nutr..

[17] Kamel D.G., Othman A.A., Osman D.M., Hammam A.R.A. (2021). Probiotic yogurt supplemented with nanopowdered eggshell: Shelf-life stability, physicochemical, and sensory characteristics. Food Sci. Nutr..

[18] Izadi Z., Nasirpour A., Garoosi G.A., Tamjidi F. (2015). Rheological and physical properties of yogurt enriched with phytosterol during storage. J. Food Sci. Technol..

[19] Ciron C.I.E., Gee V.L., Kelly A.L., Auty M.A.E. (2010). Comparison of the effects of high-pressure microfluidization and conventional homogenization of milk on particle size, water retention and texture of non-fat and low-fat yoghurts. Int. Dairy J..

[20] Fang T., Shen X., Hou J., Guo M. (2019). Effects of polymerized whey protein prepared directly from cheese whey as fat replacer on physiochemical, texture, microstructure and sensory properties of low-fat set yogurt. LWT.

[21] Sagis L.M.C., Scholten E. (2014). Complex interfaces in food: Structure and mechanical properties. Trends Food Sci. Technol..

[22] Eliseeva L.G., Kokorina D.S., Zhirkova E.V., Smirova S.A., Nevskaya E.V. (2021). The Quality and Microbiological Stability of Quinoa-enriched Wheat Bread. IOP Conf. Ser. Earth Environ. Sci..

[23] Krzeminski A., Prell K.A., Busch-Stockfisch M., Weiss J., Hinrichs J. (2014). Whey protein–pectin complexes as new texturising elements in fat-reduced yoghurt systems. Int. Dairy J..

[24] Alcântara L.A.P., Fontan R.d.C.I., Bonomo R.C.F., de Souza E.C., Sampaio V.S., Pereira R.G. (2012). Density and Dynamic Viscosity of Bovine Milk Affect by Temperature and Composition. Int. J. Food Eng..

[25] Corredig M., Dalgleish D.G. (1999). The mechanisms of the heat-induced interaction of whey proteins with casein micelles in milk. Int. Dairy J..

[26] Mottar J., Bassier A., Joniau M., Baert J. (1989). Effect of Heat-Induced Association of Whey Proteins and Casein Micelles on Yogurt Texture. J. Dairy Sci..

[27] Mahomud M.S., Katsuno N., Zhang L., Nishizu T. (2017). Physical, rheological, and microstructural properties of whey protein enriched yogurt influenced by heating the milk at different pH values. J. Food Process. Preserv..

[28] Lee W.-J., Lucey J.A. (2003). Rheological properties, whey separation, and microstructure in set-style yogurt: Effects of heating temperature and incubation temperature. J. Texture Stud..

[29] Tamime A.Y., Marshall V.M.E. (1997). Microbiology and technology of fermented milks. Microbiology and Biochemistry of Cheese and Fermented Milk.

[30] Tamime Y.A., Robinson R.K. (1999). Yoghurt-Science and Technology. Int. J. Dairy Technol..

[31] Tamime A.Y., Deeth H.C. (1980). Yogurt: Technology and Biochemistry. J. Food Prot..

[32] Gastaldi E., Lagaude A., Marchesseau S., Fuente B.T. (1997). Acid Milk Gel Formation as Affected by Total Solids Content. J. Food Sci..

[33] Cho Y.H., Lucey J.A., Singh H. (1999). Rheological properties of acid milk gels as affected by the nature of the fat globule surface material and heat treatment of milk. Int. Dairy J..

[34] Abu-Jdayil B. (2003). Modelling the time-dependent rheological behavior of semisolid foodstuffs. J. Food Eng..

[35] Damin M.R., Alcântara M.R., Nunes A.P., Oliveira M.N. (2009). Effects of milk supplementation with skim milk powder, whey protein concentrate and sodium caseinate on acidification kinetics, rheological properties and structure of nonfat stirred yogurt. LWT Food Sci. Technol..

[36] Teo C.T., Munro P.A., Singh H. (2000). Time dependence of rheological breakdown and recovery of heat precipitated whey protein suspensions. Milchwissenschaft.

[37] Barnes H.A. (1997). Thixotropy—A review. J. Nonnewton. Fluid Mech..

[38] Shaker R., Jumah R., Abu-Jdayil B. (2000). Rheological properties of plain yogurt during coagulation process: Impact of fat content and preheat treatment of milk. J. Food Eng..

[39] Kinsella J.E., Morr C.V. (1984). Milk proteins: Physicochemical and functional properties. Crit. Rev. Food Sci. Nutr..

[40] Schellhaass S., Morris H. (1985). Rheological and Scanning Electron Microscopic Examination of Skim Milk Gels Obtained by Fermenting With Ropy and Non-Ropy Strains of Lactic Acid Bacteria. Food Struct..

[41] Fangary Y.S., Barigou M., Seville J.P.K. (1999). Simulation of Yoghurt Flow and Prediction of Its End-of-Process Properties Using Rheological Measurements. Food Bioprod. Process..

[42] Lucey J.A., Teo C.T., Munro P.A., Singh H. (1997). Rheological properties at small (dynamic) and large (yield) deformations of acid gels made from heated milk. J. Dairy Res..

[43] Cruz A.G., Cadena R.S., Alvaro M.B.V.B., Sant’Ana A.S., Oliveira C.A.F., Faria J.A.F., Bolini H.M.A., Ferreira M.M.C. (2013). Assessing the use of different chemometric techniques to discriminate low-fat and full-fat yogurts. LWT Food Sci. Technol..

[44] Vélez-Ruiz J.F., Barbosa Cánovas G.V., Peleg M. (1997). Rheological properties of selected dairy products. Crit. Rev. Food Sci. Nutr..

[45] Lee W.J., Lucey J.A. (2010). Formation and Physical Properties of Yogurt. Asian J. Anim. Sci..

[46] Henriques M.H.F., Gomes D.M.G.S., Pereira C.J.D., Gil M.H.M. (2013). Effects of Liquid Whey Protein Concentrate on Functional and Sensorial Properties of Set Yogurts and Fresh Cheese. Food Bioprocess Technol..

[47] Hinrichs J. (2001). Incorporation of whey proteins in cheese. Int. Dairy J..

